# A painful experience of limited understanding: healthcare professionals’ experiences with palliative care of people with severe dementia in Norwegian nursing homes

**DOI:** 10.1186/s12904-018-0282-8

**Published:** 2018-02-13

**Authors:** May Helen Midtbust, Rigmor Einang Alnes, Eva Gjengedal, Else Lykkeslet

**Affiliations:** 1 Department for Health Sciences in Aalesund, Faculty of Medicine and Health Sciences, Norwegian University of Science and Technology, Serviceboks 17, 6025 Aalesund, NO Norway; 2 University of Bergen, Global Public Health and Primary Care, Box 7804, 5020 Bergen, Norway; 3 Molde University College, Faculty of Health Sciences and Social Care, Box 2110, 6402 Molde, Norway

**Keywords:** Palliative care, Dementia, Nursing homes, Phenomenology, Qualitative methods

## Abstract

**Background:**

People dying with dementia have significant healthcare needs, and palliative care, with its focus on comfort and quality of life, should be made available to these patients. The aim of this study was to explore and increase knowledge of healthcare professionals’ experiences with palliative care to people with severe dementia in nursing homes.

**Methods:**

To describe the phenomenon under investigation, we used a phenomenological research approach grounded in the philosophy of Husserl. Data were collected using in-depth interviews with 20 healthcare professionals from four Norwegian nursing homes.

**Results:**

The general meaning structure of the healthcare professionals’ experiences with providing palliative care to people with severe dementia is painfulness, due to their limited understanding of patients’ individual modes of expression. The painfulness is illustrated by the following themes: challenges related to “reading” the patients’ suffering, coming up short despite occasional success, handing the patients over to strangers, and disagreeing on the patients’ best interests. The healthcare professionals struggled to understand patients by “reading” their suffering. Occasionally, they succeeded and were able to calm the patients, but they often had the feeling of coming up short in situations related to pain relief and coping with behavioural symptoms, such as aggression and rejection of care. They also found it painful when the weakest patients were moved from the sheltered unit to a somatic long-term unit and were handed over to strangers who did not know the patients’ ways of expression. Although the healthcare professionals emphasized the importance of good collaboration with the patients’ relatives to ensure the best possible palliative care, they frequently found themselves in difficult situations when they disagreed with the family on the patients’ best interests.

**Conclusions:**

We found healthcare professionals’ experiences of providing palliative care to people with severe dementia to be painful. To be able to understand the patients better, long-term familiarity and knowledge of how to “read” and observe patients with severe dementia are necessary. Openness in cooperation with the patients’ relatives and with the professional team may increase healthcare professionals’ understanding of the patients’ situations and hence improve the quality of care.

## Background

Dementia is a public health challenge worldwide. In 2015, it was estimated that 47 million people were living with dementia globally, and this number is predicted to increase to 75 million in 2030 and to 132 million by 2050 [1]. Currently around 77,000 people live with dementia in Norway, and more than 80% of Norwegian nursing home residents have dementia [2]. Studies on the location of death among older people with dementia show that the majority of dementia-related deaths in the United States and Europe occur in nursing homes [3, 4]. When such a high number of people with dementia live their final phase of life in nursing homes, it is important to increase the knowledge of healthcare professionals’ experiences with caring for this patient group.

Dementia is a brain disease, often of a chronic or progressive nature, with a variety of causes. It is characterized by gradual cognitive impairment involving memory, judgement, planning, abstract thinking, and emotional and social functioning. The impairment in cognitive function leads to the deterioration of emotional control and social behaviour [5]. People with dementia experience a gradual decline, and the end stage of dementia is characterized by profound cognitive impairment, inability to communicate verbally, and complete functional dependence, often accompanied by double incontinence and dysphagia. People with advanced dementia are at increased risk of infections and are often bed- or chair-bound [6–8]. Advanced dementia has been under-recognized as a terminal illness [7, 9, 10] that requires a palliative care approach to improve quality of life [10].

Patients dying with dementia have significant healthcare needs, and palliative care should be made available to everyone, with its focus on comfort and quality of life [7, 11–13]. Communicating with people with severe dementia is difficult and presents challenges related to assessing and treating symptoms such as pain and ascertaining their wishes for end-of-life care. Understanding what provides these patients with comfort and quality of life is therefore particularly challenging. The disease trajectory for individuals with dementia is described as a period of “prolonged dwindling” [14], and several studies highlight the difficulties related to identifying a palliative phase [11, 15, 16]. A study of healthcare professionals working in long-term care settings in six European countries shows a variety of opinions regarding the point at which a person with dementia needs palliative care [15].

The inability of persons with dementia to verbally express pain and discomfort makes them a vulnerable patient group and dependent on their caregivers. Optimal palliative care includes the management of pain and symptom relief, but persons with severe dementia are at high risk of suffering from pain, as their self-report is impaired or unavailable [17–19]. Greater knowledge of pain treatment is important and requires knowledge of both pain assessment and dementia [19]. Nurses play a key role in pain management through the use of pain assessment tools and behavioural observation [18–20]. Assessing a verbally non-communicative person is difficult [12, 21], and a study on how nurses make decisions regarding symptom relief for nursing home residents with dementia at the end of life showed that decisions were triggered by the nurses’ early recognition of behavioural change. These decisions involved nuanced and detailed observations of muscle tone, facial expression and gestures that are not easily recognized by standardized assessment tools [22].

Providing palliative care to people living with advanced dementia in nursing homes is challenging [11, 12, 23]. In addition to assessment, managing physical and behavioural symptoms and communicating with the family are further challenges [12]. Communicating with patients and families of people with dementia requires special skills due to the cognitive problems that complicate decision making [10, 11]. Support for family members is important in helping them in their role as decision makers and their handling of the high burden of care and grief caused by the continuing deterioration of the patient [10]. Healthcare professionals providing palliative care for this patient group also report that they perceive themselves as experiencing a high emotional and physical burden [23]. Earlier studies note that providing palliative care to people with advanced dementia is challenging and requires special skills. Detailed descriptions of the healthcare professionals’ experiences are less available.

The aim of this study was therefore to explore and clarify healthcare professionals’ experiences with providing palliative care to people with severe dementia in nursing homes.

## Methods

To describe the phenomenon under investigation, we used a phenomenological research approach grounded in the philosophy of Husserl. Husserl’s phenomenological philosophy is based on the idea of going to the things themselves, in other words, to do full justice to the everyday experience, the lived experience, or the lifeworld. He described the lifeworld as the common, everyday world into which we are all born and live [24]. Researchers carrying out empirical research in different disciplines have developed methodologies inspired by Husserl’s phenomenology for studying empirical phenomena [25–27]. The world of experience is the core of the lifeworld research. The aim of a phenomenological lifeworld research approach is to discover, analyse, clarify, understand and describe the meaning of the phenomenon as it is experienced by the informants [25]. The focus of our project was to seek the informants’ everyday experiences with providing palliative care to people with severe dementia and to try to find the meaning of the experiences that are often implicit, tacit and taken for granted**.** It is of crucial importance to adopt a phenomenological attitude during the research process [27]. This attitude is understood as setting aside any preunderstanding of the phenomenon we are studying [27] by being reflective and open to the phenomenon and “actively waiting” for the phenomenon and its meaning(s) to emerge [28].

### Participants and recruitment

The management teams of four nursing homes in three different municipalities in mid-Norway were asked to participate in the study. For variation in the sample, two nursing homes in a mid-sized city and two nursing homes in smaller municipalities were randomly selected. The four nursing homes had 48 to 78 beds and different units, such as somatic short- and long-term wards. Three of the four nursing homes also had a sheltered ward for people with a dementia diagnosis. Sheltered units usually have fewer residents than somatic short- and long-term units, and care is adapted to patients with dementia. The managers in each nursing home were asked to recruit health personnel who might be interested in participating in the study. We included both licensed practical nurses and registered nurses because even though registered nurses have primary responsibility for palliative care in nursing homes, practical nurses have a key role in direct patient care. The practical nurses work under the direction of registered nurses. The managers gave the staff an oral description of the study and handed out a letter with the same information. Seven licensed practical nurses and 13 registered nurses, five from each nursing home, participated in the study. The healthcare professionals, all women, were employed in half-time to full-time positions, their average age was 43 years (range 28–63), and they had three to 40 years (average 18) of experience with working with people with dementia.

### Data collection

Our data gathering method was in-depth interviews with a phenomenological approach [25]. The first author conducted the interviews and sought rich and varied descriptions of healthcare professionals’ experiences. Supportive dialogue was pursued by asking open questions, giving the informant the time and space to speak without interruption, listening actively, and asking for further elaboration from the healthcare professionals when appropriate. A semi-structured interview guide was used to balance openness and focus during the interview [25]. The interview opened with the following question: What are your experiences with providing palliative care to people with severe dementia in nursing homes? The informants were encouraged to give examples from everyday work.

All interviews were carried out in suitable rooms in the nursing homes, and most interviews lasted approximately 60 min. The interviews were recorded and transcribed verbatim by the interviewer.

### Data analysis

Our analysis was inspired by Giorgi’s phenomenological method [26, 27]. First, the interview transcripts were read several times in order to grasp a sense of the overall meaning. The next step was to reread each interview and break the text into parts or meaning units related to the phenomenon under study. NVivo 11 qualitative data analysis software was used as a tool in the coding of the text. The meaning units were then grouped into subcategories related to their content. In step three, we searched for a richer and more complex lifeworld descriptions, and the meaning that was embedded in the concrete description was teased out by asking questions such as the following: What is this about? Which commonalities and differences appear? What may be the underlying meaning of the experience? The meaning units in the subcategories were then rewritten and transformed into condensed descriptions. These transformed meaning units formed the basis for step four, in which we synthesized and integrated the transformed meaning units in each interview into one consistent description. Finally, we searched for the differences and similarities across the individual descriptions, and a general structure of meaning gradually came into view. The process was one of writing and rewriting, and all authors participated in the discussions and steps of the analysis.

## Results

### A painful experience of limited understanding

The general meaning structure of the healthcare professionals’ experiences with providing palliative care to people with severe dementia is painfulness due to their limited understanding of the patients’ individual modes of expression when they are no longer able to explain their own situation. The painfulness is illustrated by the following themes: Challenges related to “reading” the patients’ suffering, coming up short despite occasional success, handing the patients over to strangers, and disagreeing on the patients’ best interests.

### Challenges related to “reading” the patients’ suffering

The healthcare professionals had all experienced that “reading” the patient and interpreting any discomfort the patient may suffer were great challenges when providing palliative care. Providing adequate care is a challenge, as patients suffering from severe dementia can no longer explain their own situation. Even though some still have language, their speech may be a medley of words that to a small or no degree describe their pain. Understanding the message and finding the cause of any pain, anxiety or discomfort demands familiarity with the patient and the way the patient expresses himself. One nurse said the following: “*There are only small signs, but just enough for those who know him well to realize that he is in pain.*” The informants described how experience had taught them to observe and examine body parts to see if this condition gave any discomfort or pain to the patient. A person with severe dementia may change their behaviour and become dismissive and aggressive in situations related to care, as well as angry and annoyed in other situations; thus, interpreting the patients’ modes of expression and behaviour may give indications of pain or other discomfort. “Reading” the patient was experienced as difficult, and it was hard to know if the interpretation was correct. One nurse said the following: “*How do we interpret? Do we interpret correctly? It is not easy to find the key. In addition, they are so different.”*

The healthcare professionals found that they must start at some end to try to determine what was troubling the patient. One nurse gave the following example: “*A patient in a sheltered unit expresses increasing uneasiness. She talks, but there is no coherence in what she says. She is angry and agitated, and her behaviour is almost aggressive. She also does not have much of an appetite. She is taken care of and examined, but health personnel are unable to figure out whether she is suffering from increasing pain or a development in the dementia*.”

Healthcare professionals in this study had all experienced that if they knew the patient well, they were able to recognize the turning points at which patients with dementia entered a new stage of their disease and approached the end of life. To be able to recognize such a turning point or any changes at all, a long-term familiarity with the patient is of paramount importance. Good cooperation with the patients’ relatives, who also know the patients’ needs and modes of expression and wishes, was also regarded as crucial to the provision of the best possible care for patients. A deteriorating health condition is often a consequence of several infections and weight loss. A patient may have repeated infections and several antibiotic treatments and still have a deteriorating health condition. One nurse said the following: “*It is like a downward spiral of infections and cures*.” The patients eat and drink less and need more help. Many patients sleep more than before, and healthcare professionals find the patient in this phase to be weary of life. Several informants described patients who close their lips tightly when offered food and drink, and even if they no longer have verbal language, they shake their heads, which may be an indication that they do not want to eat or drink. The healthcare professionals found it demanding to “read” the patients’ individual mode of expression and found it painful not to be able to understand them.

### Coming up short, despite occasional success

The healthcare professionals often experienced that working with patients with severe dementia gave them a feeling of coming up short. A practical nurse expressed the following about what was most difficult in providing palliative care to people with dementia: “*It is not understanding what’s needed. You can see, but you are not able to understand.”* Behavioura*l* symptoms, such as agitation, irritability, physical aggression and rejection of care, were challenges related to palliative care. Rejection of care is one of the most disturbing symptoms, and in their practice, taking care of personal hygiene was a recurring problem. The healthcare professionals want to help but are rejected by the patient. They described near “fights”, including beating, kicking and pinching, all painful and difficult to endure. Several healthcare professionals described witnessing a patient in great pain and then being brutally rejected, and even threatened, when trying to help, which they regarded as very troubling. One nurse said the following about such a situation: “*One minute she would say: ‘I’m going to shoot you, get out, I’m going to kill you’, and then the next minute: ‘Help me, help me, it hurts – what am I going to do?’ It was a very distressing situation both for us, for the nurses, and for the relatives*.”

Personnel found it mentally distressing when such situations kept occurring over time and perhaps several times during a shift, and this situation left the nurses feeling helpless and in despair because they really wanted to help the patient. One nurse said that no matter what she does in certain situations, it is impossible to help the patient. This experience is painful, since for her, the very essence of nursing is to be able to help and comfort.

However, in spite of many defeats, there are also bright moments when healthcare professionals feel that they succeed by coming up with measures that provide good relief for the patient. Medical relief of pain, anxiety and behavioural symptoms is important, but healthcare professionals report that comfort is also found when they sit down and hold the patient’s hand. To be calm and talk in a low voice can also be comforting, and several respondents said that familiar music can also calm patients. One nurse said that finding the right path and the “right buttons to push” can be a relief and facilitate cooperation. This practice requires time and familiarity with the person with dementia and may be a difficult challenge, but it is also very rewarding when one succeeds from time to time. A practical nurse confirmed this sentiment by describing a difficult situation with a large and physically strong man who suffered from frontal lobe dementia and had large wounds that needed care. He was aggressive and would not let anybody help him. At times, three nurses were needed to care for him. He kicked and punched, and the practical nurse described it as a nightmare. When they gradually got to know him better, they determined that he loved poems and songs. The practical nurse said that she would sometimes sing while caring for him and that succeeding in helping him felt very good.

When healthcare professionals were able to give a patient some relief from pain and anxiety and provide security for the patient and the relatives in the patient’s final phase of life, they described this experience as a feeling of success. When a patient is in a terminal phase, the healthcare professionals always spend time with him or her and provide good care for both the patient and the relatives. The relatives often express gratitude and satisfaction in the aftermath of a patient’s death, and one nurse said that situations such as these make her feel “*like she has succeeded as a nurse*”. Despite the fact that healthcare professionals occasionally experienced success in providing proper relief for a patient in the terminal phase, they are from time to time unable to provide such relief from pain and dyspnoea and to comfort anxiety and unease. Several informants had experienced that anxiety and unease can be a greater challenge than pain and that some patients remain uneasy until the end. The healthcare professionals in this study expressed their wish to make this last phase as positive as possible, and they described it as painful when they were unable to do so.

### Handing the patient over to strangers

The informants in this study who worked in sheltered units had experienced that when patients are no longer seen as needing to be in a sheltered unit, they were moved to a somatic long-term unit. The healthcare professionals listed the criteria that are taken into consideration, and most often patients are moved when they can no longer walk on their own and have to remain in a wheelchair or in bed. They felt it as painful when patients who had stayed in their unit for several years were moved. On the one hand, they understood this system, because it is practically impossible to ensure sufficient care in a sheltered unit when most patients are bedridden and need to be cared for by two nurses. A practical nurse said that at times, three out of nine patients are bedridden. With only two healthcare professionals on duty in the evenings and on weekends, there is nobody left in the common areas when the two are providing care for the bedridden patients. This situation makes the other patients uneasy because they are “alone” in the living room, and some of them start wandering around, looking for personnel. In addition, the personnel experience “pressure” from the large number of patients with dementia who are waiting for a place at a sheltered unit. Patients who are defined as no longer in need of a sheltered unit have to be removed, and their unit is given to someone else.

On the other hand, the healthcare professionals experienced moving the patient as a difficult situation for all parties. They found it painful to hand the patient over to unknown staff in a new unit that was not specifically designated for patients with dementia. Both the patient and his relatives had gotten to know the personnel in that particular unit well over time, and they now had to start all over again. Although the patients had a severe degree of dementia, the healthcare professionals found that the patients were reassured by familiar faces and voices. A patient may have spent many years in the sheltered unit before moving, and healthcare professionals had become familiar with ways to “read” the patient and interpret the signals of each patient who communicated pain and discomfort.

Even though patients are commonly moved when they become bedridden or in need of a wheelchair, the informants in this study had all experienced having patients who spent their final days in a safe and familiar environment in their unit. A patient who suffers from pneumonia, a stroke or a fractured bone is not moved if his or her life expectancy is short. Several of the healthcare professionals also said that they sometimes had to argue against the head of the unit in order to make exceptions to the rule. One nurse described a situation in which the staff fought *“tooth and nail”* to give a woman who had stayed in the unit for almost ten years the possibility of spending her final days there. According to procedure, the patient should have been moved a long time ago, but she was so attached to the personnel and so anxious that the management gave in and let her stay in the unit during her final days. In some situations, the healthcare professionals find that the relatives fight to have their loved ones remain in the unit. All participants in this study had experienced “strong relatives” attempting to have their family member spend the remaining days of their lives in safe and familiar surroundings. Healthcare professionals reported that even though the relatives had been informed about a move, they refused to give up hope that their family member would be able to remain in the same familiar unit. One nurse described a 99-year-old patient who returned from the hospital to the sheltered unit after a fracture. The daughter was very worried that her mother would be transferred to another unit, as she knew this occurrence to be normal practice. When she was confident that her mother would not be moved, she expressed great relief that her mother had the opportunity to spend the final days of her life in a safe environment with familiar nurses. The nurse said that it would almost have been an assault to move her when she had such a short life expectancy and added the following: “*Yes, she got the chance to die here, and the relatives were incredibly happy, and so were we.”*

### Disagreeing on the patients’ best interests

All informants emphasized the importance of good cooperation with relatives to ensure the best possible palliative care. When the family agreed to palliative care in the final phase of the patient’s life, the goal was to promote physical and psychological well-being. Burdensome interventions, such as tube feeding or intravenous nutrition, were rarely implemented. However, they frequently found themselves in difficult situations, with healthcare professionals and relatives disagreeing and holding different understandings of patients’ best interests in the terminal phase. Several informants reported that relatives might be angry, frustrated and scared, and good cooperation could be difficult to attain. A frequent problem is that a patient is no longer able to eat or drink. The healthcare professionals then found that the relatives were afraid that their loved ones would suffer from hunger or thirst and would demand tube nutrition and intravenous fluids. Despite both the doctor and the nurse explaining to the relatives what occurs in the body with fluid retention in the lungs and the discomfort it may cause, the relatives insisted that the treatment continue. Several informants referred to these situations as an ethical dilemma because they see a patient in great pain and gurgling, and they know that giving intravenous fluids may cause more suffering for the patient and prolong the terminal phase. At the same time, they must be understanding towards the relatives and try to keep “calm”. One nurse said, “*Does this benefit the patient, or is it in fact the relatives we take into account? This is an ongoing dilemma, and a very difficult one*.”

In other situations, the healthcare professionals had patients with recurring infections and relatives who wanted to continue treatment when healthcare professionals thought this course of action was no longer in the patient’s best interest. One nurse said that the relatives could not see how much this treatment affected the patient since they only visited from time to time, whereas the staff in the unit knew the patient well and see his deterioration, with increasing pain and less food intake. When the nurse is no longer able to find an artery for the cannula, the next step is to give antibiotics as an intramuscular injection to a patient who does not understand why he is being injected. The healthcare professionals found these situations difficult because they felt forced to give painful treatment to a terminal patient who, in their opinion, should be spared such treatment. The relatives might say it was just a little prick, but the healthcare professionals found this far more difficult with patients with severe dementia who get scared, uneasy and show no understanding of why they are being pricked. One nurse said, “*It is never good to look someone in the eyes when they are afraid and wonder what happens*.”

## Discussion

The purpose of this study was to explore and increase knowledge of healthcare professionals’ experiences with palliative care for people with severe dementia in nursing homes. The major findings indicate that healthcare professionals experience it as painful to have limited understanding of the patients’ individual modes of expression when the patients are no longer able to verbally express their needs. The healthcare professionals struggled to understand how to help. Occasionally they succeeded, but quite often they had the feeling of coming up short in situations related to pain relief and to coping with behavioural symptoms such as aggression and rejection of care. Further, the healthcare professionals experienced it as even more painful when the weakest patients were moved to a new unit and handed over to strangers who did not know the patients’ ways of expressing themselves and therefore had less ability to understand their symptoms. Similarly, the healthcare professionals found it difficult when they disagreed with relatives on the patients’ best interest. A hermeneutic perspective on understanding may contribute to greater insight into the different aspects of our findings.

The circle of understanding is possibly the best-known term in hermeneutics and refers to a principle of interpretation stating that we understand the part from the whole and the whole from the parts [29, 30]. In our study, this concept indicates that the patients’ behaviour and expressions must be seen in light of a larger whole, in which the patients’ history and social context are significant. According to hermeneutics, all people are embedded in their own history and culture, which shape them and form a “horizon” from which new knowledge emerges. Therefore, we cannot understand any given situation without a horizon or some form of preunderstanding related to history, culture, previous knowledge and experiences. Our horizon is as far as we can see or understand, and an expanded understanding emerges when our present horizon is moved to a higher level. This process is referred to as a “fusion of horizons”, where a fusion of old and new understanding creates a new meaningful whole. To gain insight into others’ lifeworld, one must be willing to correct one’s own prejudices or put them at risk, as Gadamer puts it [29]. This process may be challenging when facing experiences or parts that at first do not make sense or agree with our preunderstanding. It is important, then, to emphasize that while our previous horizon is crucial for new understanding, it may also prevent such understanding. By attempting to understand another person, our attention must be directed towards the other, meaning that being open and trying to look beyond one’s own horizon is a significant prerequisite for gaining new understanding [29, 30] and is crucial in a phenomenological lifeworld approach.

Healthcare professionals who have known patients with dementia over time and who strive for openness are in many situations well equipped to understand the patients’ expressions even when their behaviour changes. Although long experience and openness are prerequisites for understanding the patients’ situations, they are rarely enough. The experience must be based on knowledge about palliative care and dementia. Improving these patients’ symptoms, respecting the dignity and thus maintaining the quality of life for both patients and families are essential. Experience and knowledge of how to perform close observation may enable healthcare professionals in many situations to understand that patients are in pain or discomfort by “reading” and interpreting changes in, for example, facial expression or behaviour. Such signs may be small but are just enough for those who have experiences with similar situations and know the patient well enough to understand that he is in pain or has entered a new stage of the disease. Healthcare professionals may also be able to relieve the patient from pain and anxiety and provide safety in the final phase of the patient’s life. The need for close observation and long familiarity with individuals with dementia has also been confirmed in earlier studies [12, 17, 22, 31]. Cooperation with family members, who also know the patients’ needs, wishes and ways of expression, can partially compensate for the healthcare professionals’ limited understanding. Close cooperation between the professional team and the patients’ relatives may also contribute to the expansion of the healthcare professionals’ horizons and thus their understanding.

However, when healthcare professionals found it challenging at times to understand persons with severe dementia, this situation required that they remain open to searching for a new and meaningful whole. Pain, aggression and rejection of care among persons with severe dementia are described as difficult in palliative care in this and other studies [9, 23, 32, 33]. The healthcare professionals wanted to help and experienced it as painful to witness a patient being aggressive or in great pain without being able to understand how to help. The healthcare professionals were unable to enter into the patient’s horizon, and the patient was unable to inform about his situation. Pain does not only strike when the healthcare professionals do not understand the patient’s expressions. It is also painful to leave the patient with strangers towards the end of the patient’s life. The paradox is that the patients are moved when they are so weak and ill that they are more than ever in need of familiar healthcare professionals. The healthcare professionals who do not know the patient are even worse off in understanding him, and they may not have the preunderstanding necessary to expand their own horizons.

Although the informants in this study emphasized the importance of good collaboration with relatives to ensure the best possible palliative care, they quite often found themselves in situations in which they disagreed and had a different understanding of the patients’ best interests. The disagreement was frequently related to issues such as tube nutrition and intravenous fluids or antibiotics, and the healthcare professionals felt forced by the family to give painful treatment to a terminal patient. Infections and eating problems are frequent complications in advanced dementia and are associated with high mortality and burdensome interventions [9, 11, 34]. The different understandings of the patients’ best interests may be related to poor communication with the family about end-of-life issues and the family’s role as a party in decision-making processes when the patients can no longer express their wishes. In accordance with hermeneutics theory, the healthcare professional and the family may have separate horizons in such situations. Both understand something the other does not understand. Therefore, healthcare professionals providing palliative care to people with dementia must pay attention to the patients’ families and support and help them in their roles as decision makers. The relatives should be actively involved in the decision-making process and be informed in detail about the benefits and burdens of different treatment options [7, 10]. However, the healthcare professional should also listen openly and humbly to the relatives’ knowledge. Such cooperation may contribute to a fusion of horizons and the arrival at a common understanding of the patients’ best interests and promote dignity and quality of life for both patients and their families in the final phase of life.

### Strengths and limitations

Our study may, to some extent, provide a true picture of the variation in Norwegian nursing homes. This variation is due to our inclusion of healthcare professionals with different levels of education working in different units: somatic short- and long-term wards and sheltered wards for people with dementia from four different nursing homes. We also consider our close cooperation and reflection in the research group through all stages of the research process as a strength in this study.

Some limitations should be acknowledged, especially the fact that the information given to the informants about the project before the interviews was of varying quality. The management team in each nursing home was asked to recruit healthcare professionals, give them oral information and hand out a letter with information about the study. Despite this process, it seemed that some of the informants had not received enough information about the project and the opportunity to think things through and prepare for the interviews. In addition, the recruitment of healthcare professionals to the interviews may have been affected and controlled by the managers in the nursing homes who were asked to recruit informants who might be interested in participating in the study. The managers may have chosen informants they thought were suitable and thus excluded others who might add important information to the study.

## Conclusion

This study highlights the challenges healthcare professionals experience in palliative care for people with severe dementia in nursing homes. Persons suffering from severe dementia are no longer able to verbally express themselves, which makes them a vulnerable patient group and dependent on their caregivers. The goal of palliative care is to promote physical and psychological well-being in order to improve quality of life for patients and their families. However, healthcare professionals struggle to understand how to help persons with severe dementia. The professionals experienced such limited understanding as painful, and they often had the feeling of coming up short in situations related to pain relief and to coping with behavioural symptoms, such as aggression and rejection of care. For healthcare professionals to be able to understand the patients’ various expressions, long-term familiarity with and knowledge of how to “read” and observe a person with severe dementia are necessary. Close cooperation within the professional team and with the patients’ families may also contribute to the expansion of the healthcare professionals’ horizons and thus their understanding.

Our findings indicate a need for further research on how healthcare professions can provide high quality of palliative care to patients with severe dementia in nursing homes. Further research on healthcare professionals’ experiences with barriers and facilitators of palliative care would hence be useful. Greater understanding about how to address the applicability of palliative care is important in relation to the focus on comfort and quality of the last stages of life. Close cooperation with the family is a core area in palliative care, and further research on cooperation and shared decision-making is needed from the perspectives of both healthcare professionals and family caregivers. Related to findings in this study, it would be interesting to learn more about how prepared healthcare professionals feel in caring for someone living with dementia and to explore this theme specifically in terms of palliative care.
